# The Construction of Suersen’s Obturators

**Published:** 1885-05

**Authors:** Th. Weber

**Affiliations:** Helsingfors, Finland


### THE CONSTRUCTION OF SUERSEN'S OBTURATORS.

BY DR. TH. WEBER, HELSINGFORS, FINLAND.

(Continued from page 190, April number.)

The advantages of Suersen's hard rubber, or metal obturators over others, will be acknowledged by any impartial observer who comprehends the various methods of making them. From Dr. N. W. Kingsley's Oral Deformities, I quote the following:

"The sum of my experience with obturators is this: 1st. That of all obturators this (referring to an obturator constructed entirely of metal) is the best form for a congenital fissure; but while the wearer is enabled to articulate perfectly with such an instrument, it is only after he has learned articulation with another apparatus." "2d. That a soft, elastic artificial velum is much better adapted to the acquirement of articulation than one of an unyielding, non-elastic substance, but when acquired an obturator may be substituted." "3d. That in very rare cases articulation may be acquired with an obturator only, but it is the result of the extra activity of the pharyngeal muscles, while with the elastic velum the levators of the palate contribute largely."

Although I have the greatest respect for the experience of Dr. N. W. Kingsley, yet I claim that with either a hard rubber or metallic instrument, constructed after Suersen's principles, a patient will learn to articulate just as readily as with an elastic velum. Dr. Kingsley, at the meeting of the First District Dental Society for February, 1885, remarked that no one could construct an obturator for a patient with a congenital cleft, in which articulation was noticeably improved during the first month's wear. At the March clinic of the same society, I exhibited a patient who had worn a Suersen's apparatus about three weeks, the bulb of which was made of gutta percha, and yet the improvement in articulation was quite perceptible when the patient read with the obturator. Why, then, should we make a patient wear an elastic velum, when articulation can be acquired with a more permanent instrument, which certainly is easier to construct than those of soft rubber, unless the practitioner has a large variety of the various shapes and sizes of metallic moulds from which one could be selected to fit almost any case?

Dr. Suersen, up to May, 1877, had supplied two hundred and forty-three patients with obturators, about two-thirds of whom were congenitally afflicted, and every one, so far as I know, has acquired articulation, and many even to the degree that detection of an imperfection in the vocal organs is impossible. One of these patients was a young man about fourteen years of age, when the obturator was inserted. He afterwards studied theology and became a minister.

A young lady patient, on account of her bad articulation before the insertion of the obturator, was absolutely unintelligible to strangers. She has now become a teacher of the German language, in England.

Another lady, who was engaged to be married, did not tell her intended husband of her infirmity until the day before the marriage, and the gentleman afterwards told Dr. Suersen that he had not, during the time of their engagement, noticed anything abnormal in her speech.

Another young lady, who had been married three years, assured Dr. Suersen that her husband had no knowledge of her wearing an obturator.

None of these patients had ever worn any other apparatus than Suersen's, made entirely of hard rubber, which demonstrates that perfect articulation may be acquired by their use, without the previous wearing of any other apparatus. The construction of these obturators, especially when made entirely of hard rubber, is quite simple, and the first part of it is identical with the making of an ordinary hard rubber plate. A perfect impression should be obtained with plaster of paris. In no instance is it necessary to take it further down than to the anterior border of the soft palate, nor in cases where the hard palate is perforated, higher up than the floor of the nasal cavities. To prevent the plaster from entering this cavity I pack it full of cotton, previously moistened with a solution of salt water. When this is done, the taking of the impression for an obturator is as simple as for an artificial upper plate.

(TO BE CONTINUED.)

